# Plasma Extracellular Vesicle Surface Marker Profiling Reveals Immune Cell–Associated Mitochondrial Membrane Potential Alterations in Long COVID and Myalgic Encephalomyelitis/Chronic Fatigue Syndrome

**DOI:** 10.1093/ofid/ofag209

**Published:** 2026-05-12

**Authors:** Gentaro Ikeda, Mariko Koike-Ieki, Hiroyuki Inoue, Arya V Dadhania, Vanessa El Kamari, Prasanna Jagannathan, Linda N Geng, Mitchell G Miglis, Robert W Shafer, Phillip C Yang, Hector Fabio Bonilla

**Affiliations:** Stanford Cardiovascular Institute and Division of Cardiovascular Medicine, Department of Medicine, Stanford University School of Medicine, Stanford, California, USA; Stanford Cardiovascular Institute and Division of Cardiovascular Medicine, Department of Medicine, Stanford University School of Medicine, Stanford, California, USA; Stanford Cardiovascular Institute and Division of Cardiovascular Medicine, Department of Medicine, Stanford University School of Medicine, Stanford, California, USA; Division of Infectious Diseases and Geographic Medicine, Department of Medicine, Stanford University School of Medicine, Stanford, California, USA; Division of Infectious Diseases and Geographic Medicine, Department of Medicine, Stanford University School of Medicine, Stanford, California, USA; Division of Infectious Diseases and Geographic Medicine, Department of Medicine, Stanford University School of Medicine, Stanford, California, USA; Division of Primary Care Population Health, Department of Medicine, Stanford University School of Medicine, Stanford, California, USA; Department of Neurology and Neurological Science, Stanford University School of Medicine, Stanford, California, USA; Division of Infectious Diseases and Geographic Medicine, Department of Medicine, Stanford University School of Medicine, Stanford, California, USA; Stanford Cardiovascular Institute and Division of Cardiovascular Medicine, Department of Medicine, Stanford University School of Medicine, Stanford, California, USA; Division of Infectious Diseases and Geographic Medicine, Department of Medicine, Stanford University School of Medicine, Stanford, California, USA

**Keywords:** Long COVID, Myalgic Encephalomyelitis/Chronic Fatigue Syndrome, extracellular vesicles, Biomarker

## Abstract

**Background:**

Long COVID (LC) is characterized by symptoms persisting at least 3 months after SARS-CoV-2 infection and affecting multiple organ systems. Diagnosis relies on subjective criteria without established biomarkers. Immune dysregulation and mitochondrial dysfunction are implicated in LC pathophysiology. Given clinical overlap with myalgic encephalomyelitis/chronic fatigue syndrome (ME/CFS), we investigated whether plasma extracellular vesicles (EVs) capture shared molecular signatures.

**Methods:**

Plasma EVs from 125 individuals across pandemic-era and prepandemic cohorts were analyzed. The pandemic-era cohort included COVID-Recovered, LC with ME/CFS phenotype (LC-ME/CFS), and ME/CFS without infection (pan-ME/CFS). The prepandemic cohort included ME/CFS and matched controls. Extracellular vesicles were isolated using size-exclusion chromatography. Concentration and size were assessed by nanoparticle tracking analysis, and surface markers and mitochondrial membrane potential were evaluated by flow cytometry.

**Results:**

Both pan-ME/CFS and LC-ME/CFS exhibited elevated EV concentrations compared with COVID-recovered controls after false discovery rate (FDR) correction (*q* = 0.0042 and 0.0024). Leukocyte-, monocyte/macrophage-, and platelet-derived EVs were increased, whereas B cell–derived EVs were reduced in both groups. Compared with controls, pan-ME/CFS demonstrated increased mitochondrial membrane potential in B cell–, monocyte/macrophage-, and NK cell–derived subsets after FDR correction, whereas no significant differences were observed in LC-ME/CFS. Prepandemic ME/CFS showed a nominal increase in leukocyte-derived EVs that did not persist after correction, whereas elevated mitochondrial membrane potential in B cell–derived EV subsets remained significant.

**Conclusions:**

ME/CFS and LC-ME/CFS demonstrate partially overlapping immune cell–associated EV alterations. Mitochondrial membrane potential alterations within selected immune-derived EV subsets, particularly B cell–associated EVs, suggest immune-metabolic involvement. Plasma EV profiling may inform future biomarker development.

## Teaser

Plasma extracellular vesicles in myalgic encephalomyelitis/chronic fatigue syndrome and long COVID exhibit enrichment of mitochondrial membrane potential-positive signals within immune cell–derived subsets, highlighting shared immune-metabolic alterations and supporting their potential as candidate biomarkers.

Long COVID (LC) is characterized by a heterogeneous constellation of symptoms following SARS-CoV-2 infection, leading to marked reductions in quality of life and placing a considerable burden on global healthcare systems. The estimated societal costs range from at least $2.01 to as much as $6.56 billion [1]. The cumulative global incidence of LC has been estimated at 65 million in 2020, 211 million in 2021, 337 million in 2022, and 409 million in 2023 [2]. A recent meta-analysis has identified risk factors including female sex, age ≥40 years, and smoking, while COVID-19 vaccination is protective [3]. Although the underlying mechanisms of LC remain unclear, proposed contributors include immune dysregulation, viral persistence, procoagulatory effects, endothelial dysfunction, and microcirculatory disturbances [4]. Accumulating evidence suggests that mitochondrial dysfunction is central to the pathogenesis of LC [5–7]. Peripheral blood mononuclear cells from affected individuals exhibit abnormalities in mitochondrial respiration, bioenergetics, and gene expression [8–10], while magnetic resonance spectroscopy has demonstrated impaired energetics in both muscle and brain tissues [11–14].

Myalgic encephalomyelitis/chronic fatigue syndrome (ME/CFS) is a debilitating disorder characterized by profound fatigue, postexertional malaise, unrefreshed sleep, cognitive dysfunction, and orthostatic intolerance, affecting up to 2.5 million individuals in the United States prior to the pandemic [15]. Recent studies indicate that approximately half of patients with LC meet ME/CFS diagnostic criteria, suggesting that COVID-19 infection can trigger an ME/CFS phenotype similar to that observed following other viral infections [16]. Similar to LC, the etiology of ME/CFS has been linked to immune dysregulation and mitochondrial dysfunction, including impaired NK cell activity [17–19], T-cell exhaustion [20–22], redox imbalance [7, 23], and disrupted mitochondrial metabolism [24–27]. These findings parallel mitochondrial abnormalities reported in LC [5–14], suggesting shared pathogenic pathways. Despite these insights, diagnosis of both LC and ME/CFS still relies on subjective clinical criteria, underscoring the urgent need for objective biomarkers to enable accurate diagnosis and patient stratification. Importantly, only a limited number of clinical studies have directly evaluated mitochondrial dysfunction in patients with LC [7].

Extracellular vesicles (EVs) have emerged as key mediators of intercellular communication and potential biomarkers of disease [28]. Extracellular vesicles are lipid bilayer particles released by virtually all cell types, carrying proteins, nucleic acids, and organelles that reflect the physiological state of their cells of origin [29, 30]. Extracellular vesicles can be readily assessed from peripheral blood, making them attractive candidates for noninvasive biomarkers or “liquid biopsies.” Importantly, recent studies have identified subsets of EVs enriched with mitochondria, which retain membrane potential and modulate bioenergetics in recipient cells [31–35]. However, the role of mitochondria-positive EVs in LC and ME/CFS remains largely unexplored.

In this study, we investigated plasma EVs from both prepandemic and pandemic-era cohorts. The pandemic-era cohort included individuals with ME/CFS, LC-ME/CFS, and COVID-recovered controls, to identify shared and distinct EV characteristics. We assessed EV concentration, cellular origin, and mitochondrial properties using flow cytometry and nanoparticle tracking analysis. To validate ME/CFS-specific EV alterations independent of prior COVID-19 infection or vaccination, we examined prepandemic ME/CFS samples in comparison with matched healthy controls.

## METHODS

### Participant Selection and Evaluation

**Figure 1.**
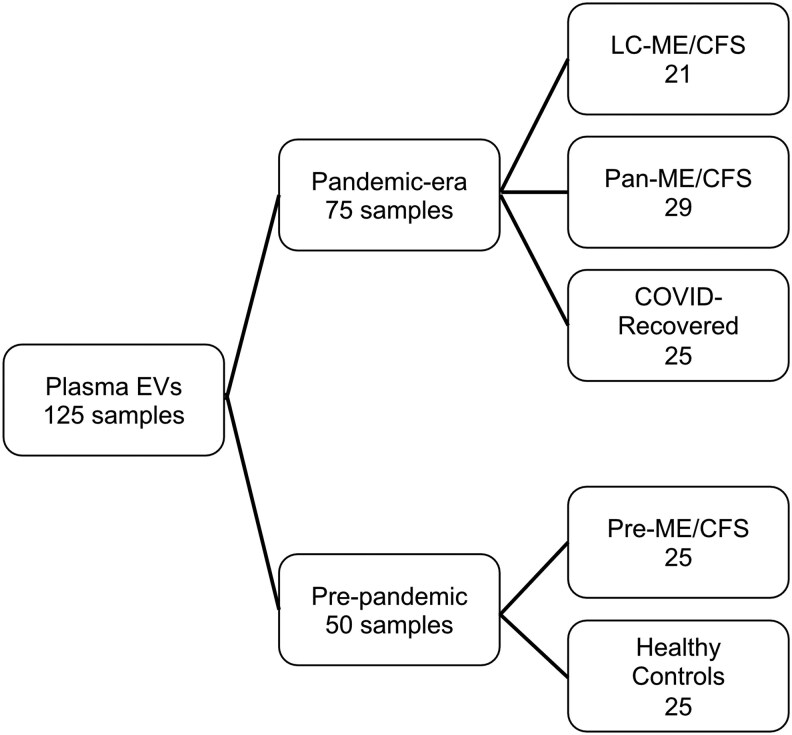
Clinical cohorts and sample distribution used for extracellular vesicle (EV) analysis. A total of 125 plasma EV samples were analyzed, comprising 75 pandemic-era and 50 prepandemic samples. Pandemic-era samples included individuals with long COVID and myalgic encephalomyelitis/chronic fatigue syndrome (ME/CFS) phenotype (LC-ME/CFS, n = 21), ME/CFS without SARS-CoV-2 infection (pan-ME/CFS, n = 29), and recovered COVID-19 patients without ME/CFS (COVID-recovered, n = 25). Prepandemic samples were obtained from individuals with the pre-ME/CFS enrolled in a 2009 study (n = 25) and age-matched healthy controls (n = 25).

All procedures involving human participants were approved by the Stanford University Institutional Review Board, and all participants provided written informed consent. We analyzed 125 heparin plasma EV samples collected from 125 individuals (Figure 1, Table 1). Samples were derived from 2 temporally distinct cohorts: a pandemic-era cohort and a prepandemic cohort.

**Table 1.** COVID-19 Positivity Reflects a Documented Prior SARS-CoV-2 Infection Confirmed by Reverse Transcription Polymerase Chain Reaction or Antigen Testing

Cohort	n	ME/CFS Diagnosis	Prior SARS-CoV-2 Infection n (%)	COVID-19 Vaccination n (%)	Mean Age Years (Range)	Female n (%)
Pandemic-era						
LC-ME/CFS	21	Yes	21 (100)	11 (52)	48.0 (22–67)	18 (85)
Pan-ME/CFS	29	Yes	3 (10)	17 (58)	43.5 (21–69)	22 (75)
COVID-recovered	25	No	25 (100)	0 (0)	41.0 (21–63)	6 (24)
Prepandemic						
Pre-ME/CFS	25	Yes	Not applicable	Not applicable	Not available	Not available
Healthy controls	25	No	Not applicable	Not applicable	Not available	Not available
Total	125					

No samples were collected during acute SARS-CoV-2 infection.

The pandemic-era cohort included 75 samples: 25 from COVID-recovered individuals (collected from 4 February 2021 to 1 May 2021), 21 from individuals with an LC-ME/CFS phenotype, and 29 from individuals with ME/CFS (collected from 27 April 2022 to 2 November 2023). For the COVID-recovered and LC-ME/CFS groups, SARS-CoV-2 infection was confirmed by reverse transcription polymerase chain reaction or antigen positivity. All samples from these 2 groups were collected after recovery from SARS-CoV-2 infection and not during acute COVID-19.

The LC-ME/CFS samples were from participants with confirmed SARS-CoV-2 infection and an ME/CFS phenotype diagnosed according to the Institute of Medicine (IOM) criteria [36]. In this group, 48% had not received COVID-19 vaccination, the mean age was 48 years, 85% were female, and the median duration of persistent symptoms was 541.5 days (range, 96–994). The ME/CFS samples were obtained from participants who had been clinically diagnosed with ME/CFS prior to the COVID-19 pandemic using the Fukuda CDC [37], IOM [36], and Revised Canadian Consensus Criteria [38], as part of the Stanford ME/CFS clinic cohort. The precise etiology of the condition could not be definitively established, and because plasma samples were collected during the pandemic period, the potential influence of SARS-CoV-2 infection or other pandemic-related factors on disease course or underlying biology, including asymptomatic or unrecognized infection, cannot be excluded. Testing for nonspike SARS-CoV-2 antigens was not performed. In this subgroup, 3 participants (10%) had a documented history of prior SARS-CoV-2 infection; however, all had recovered and were not COVID-19 positive at the time of blood draw. In addition, 58% were vaccinated, 75% were female, and the mean age was 43 years. The remaining 25 samples were obtained from the Stanford Lambda trial, which enrolled individuals with mild-to-moderate acute SARS-CoV-2 infection who underwent 10-month longitudinal follow-up and fully recovered (ClinicalTrials.gov NCT04331899) [39]. In this cohort, none were vaccinated (0%), 24% were female, and the mean age was 41 years. All plasma samples from this cohort were collected after recovery from acute infection.

The prepandemic cohort consisted of 50 samples derived from the Stanford Cytokines in ME/CFS study (2009), including 25 participants with ME/CFS (diagnosed according to the Fukuda CDC criteria) and 25 age- and sex-matched healthy controls. The reported demographic characteristics of the parent cohort were a mean age of 49.9 years for patients with ME/CFS and 50.1 years for healthy controls, with 76% and 77% female participants, respectively [40]. Demographic information specific to the samples analyzed in the present study was not available. Blood was collected in heparin tubes, centrifuged within 1 hour of collection at 1800*g* for 15 minutes, and cryopreserved at −80 °C.

### Isolation of Plasma EVs

A 0.5-mL aliquot of thawed plasma was centrifuged at 1500*g* for 10 minutes at 4 ℃. Extracellular vesicles ranging from 70 to 1000 nm were isolated using size-exclusion chromatography (qEVoriginal/70 nm Gen 2 column, Izon Science), according to the manufacturer's instructions. This size range was selected to encompass larger vesicular populations enriched in mitochondrial components [31, 32]. Columns were equilibrated with 20 mL sterile-filtered phosphate-buffered saline (PBS) at room temperature. Five hundred microliters of plasma sample were loaded onto the column, and initial 3.0 mL void volume was discarded. The subsequent 1.5 mL EV-enriched fraction was collected and immediately stored at −80 °C until analysis. To control for potential column contamination, a PBS-only sample was processed in parallel as a negative control.

### Nanoparticle Tracking Analysis

Size distribution and concentration of EVs were evaluated by nanoparticle tracking analysis (NTA) using a NanoSight LM20 (Malvern Panalytical, United Kingdom). Prior to measurement, all EV samples were diluted 1:10 in sterile-filtered PBS to achieve optimal particle concentration within the recommended detection range. Diluted samples were loaded into the sample chamber with sterile syringes and analyzed using a 40 mW 640 nm laser. For each sample, three 60-second videos were acquired under consistent camera level and detection threshold settings. Data were analyzed using NTA software, and the mean particle size and concentration across the 3 recordings were reported.

### Flow Cytometry-Based EV Analysis

Flow cytometry was performed at the Stanford Shared FACS Facility using a BD LSRII cytometer. Sample preparation followed previously established protocol [31–33, 41]. Samples were stained with pretitrated volumes of the following fluorochrome-conjugated monoclonal antibodies and incubated at 4 °C for 30 minutes: CD3 (T cell)-BV510, CD20 (B cell)-BUV805, CD45 (leukocyte)-PerCP-Cy5.5, CD14 (monocyte/macrophage)-SuperBright780, CD144 (endothelial cell)-PE-Vio770, CD16-BV711 (NK cell), CD41a (platelet)-PE-Cy5, and CD56 (neural cell adhesion molecule, NCAM)-Fluorescein isothiocyanate. To evaluate nonspecific binding, fluorochrome- and isotype-matched control antibodies were included. Detailed information regarding antibody clones, isotypes, and catalog numbers is provided in Supplementary Table 1. Additionally, 100 nM MitoTracker Deep Red (MTDR, Thermo Fisher Scientific) was used. Prior to use, all antibodies were filtered through 0.22 μm centrifugal filters (Ultrafree MC-GV centrifugal filters, Millipore) to remove aggregates. Control included PBS-only, PBS with reagents, and unstained samples, all analyzed under the same cytometer settings as the experimental samples, including triggering threshold, voltages, and flow rates. Unstained controls were prepared at the same dilution as stained samples. The event rate for PBS-only controls was maintained below 1500 events/second. The instrument was calibrated using PBS-only control and size-standard beads (Apogee Flow Systems). Data acquisition was triggered on side scatter at a threshold set between 220 and 250 arbitrary units. Flow rate was monitored using counting beads, and fluorescence calibration was performed with Rainbow Fluorescent Particles (Spherotech Inc., IL). Acquisition settings, including triggering threshold, voltages, and flow rates, were maintained for all samples.

**Figure 2.**
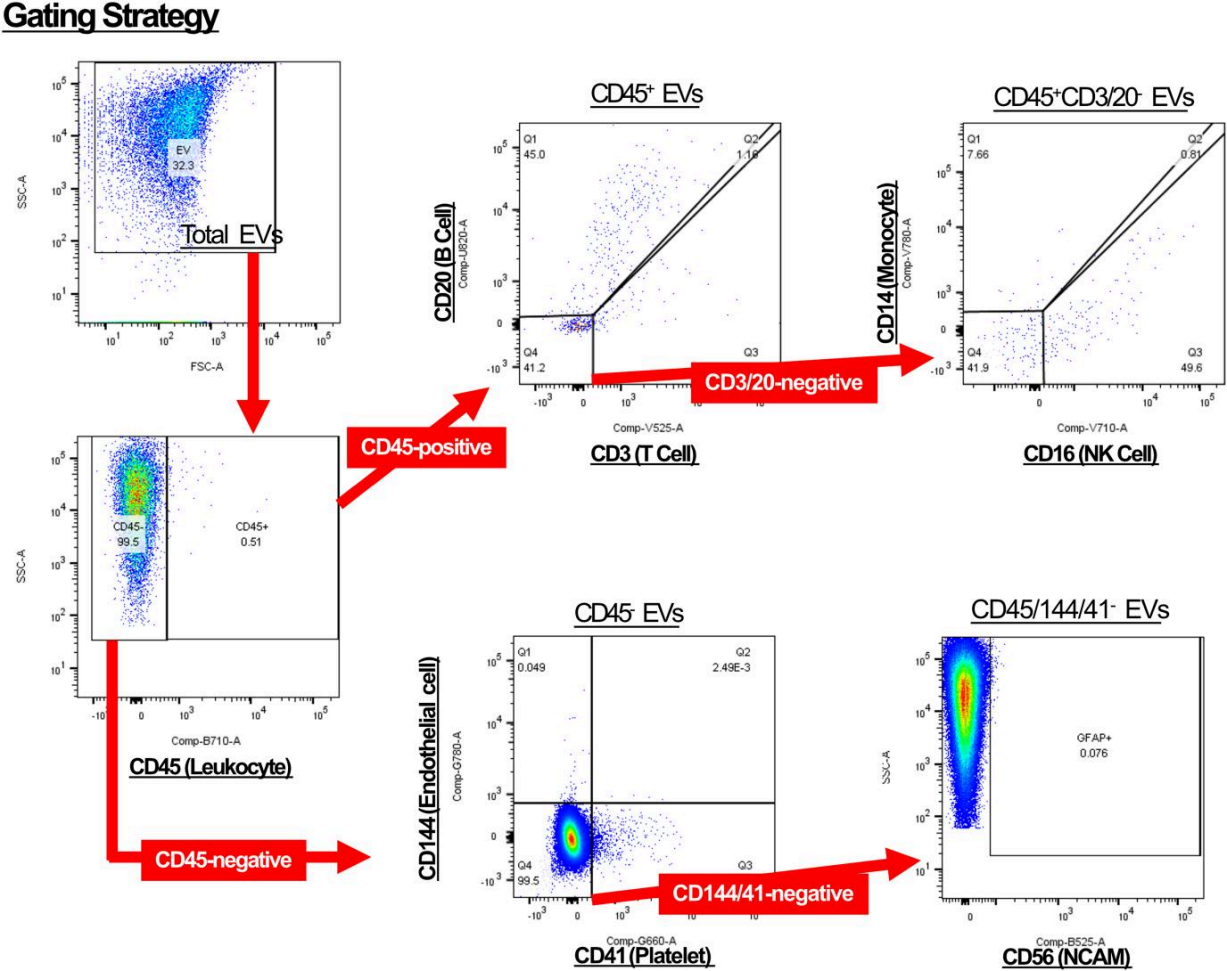
Gating strategy for flow cytometric analysis of plasma extracellular vesicles (EVs) by cellular origin. Plasma EVs were first identified based on their forward and side scatter properties. Using CD45 staining, EVs were separated into CD45-positive and CD45-negative populations. CD45-positive EVs, representing leukocyte-derived vesicles, were further subdivided based on specific surface markers to identify EVs derived from T cells (CD3^+^CD20^−^), B cells (CD3^−^CD20^+^), monocytes (CD3^−^CD20^−^CD14^+^), and natural killer (NK) cells (CD3^−^CD20^−^CD16^+^). CD45-negative EVs were gated to identify nonleukocyte-derived vesicles, including endothelial-derived EVs (CD144^+^), platelet-derived EVs (CD41^+^), and neural-derived EVs (CD144^−^CD41^−^CD56^+^). The gating strategy was applied sequentially as indicated by the arrows.

Data were analyzed using FlowJo software (BD Biosciences, OR), with the gating strategy shown in Figure 2. Extracellular vesicle populations were identified based on side scatter area (SSC-A) characteristics, with a threshold set at the SSC-A value corresponding to 110 nm standard beads. Extracellular vesicles of inflammatory origin were classified as follows: total leukocyte-derived EVs (CD45^+^), B cell derived (CD45^+^CD20^+^), T cell derived (CD45^+^CD3^+^), monocyte/macrophage derived (CD45^+^CD20^−^CD3^−^CD14^+^), and NK cell derived (CD45^+^CD20^−^CD3^−^CD16^+^). Noninflammatory EVs were defined as endothelial derived (CD45^−^CD41^−^CD144^+^), platelet derived (CD45^−^CD144^−^CD41^+^), and neural cell derived (CD45^−^CD144^−^CD41^−^CD56^+^). Although CD56 (NCAM) is also expressed on NK cells [42], it has been used as a neural-associated marker in plasma EV studies [43, 44]. Therefore, CD56 positivity was interpreted within the CD45^−^ fraction to minimize hematopoietic contribution, and CD45^−^CD56^+^ EVs were considered neural enriched rather than neural specific. Gating was based on the negative controls and isotype controls. To ensure consistency across experiments performed on different days, a common reference sample was included in each session (5 samples/session), confirming reproducibility of marker expression across sessions.

### Mitochondrial Membrane Potential in EVs

**Figure 3.**
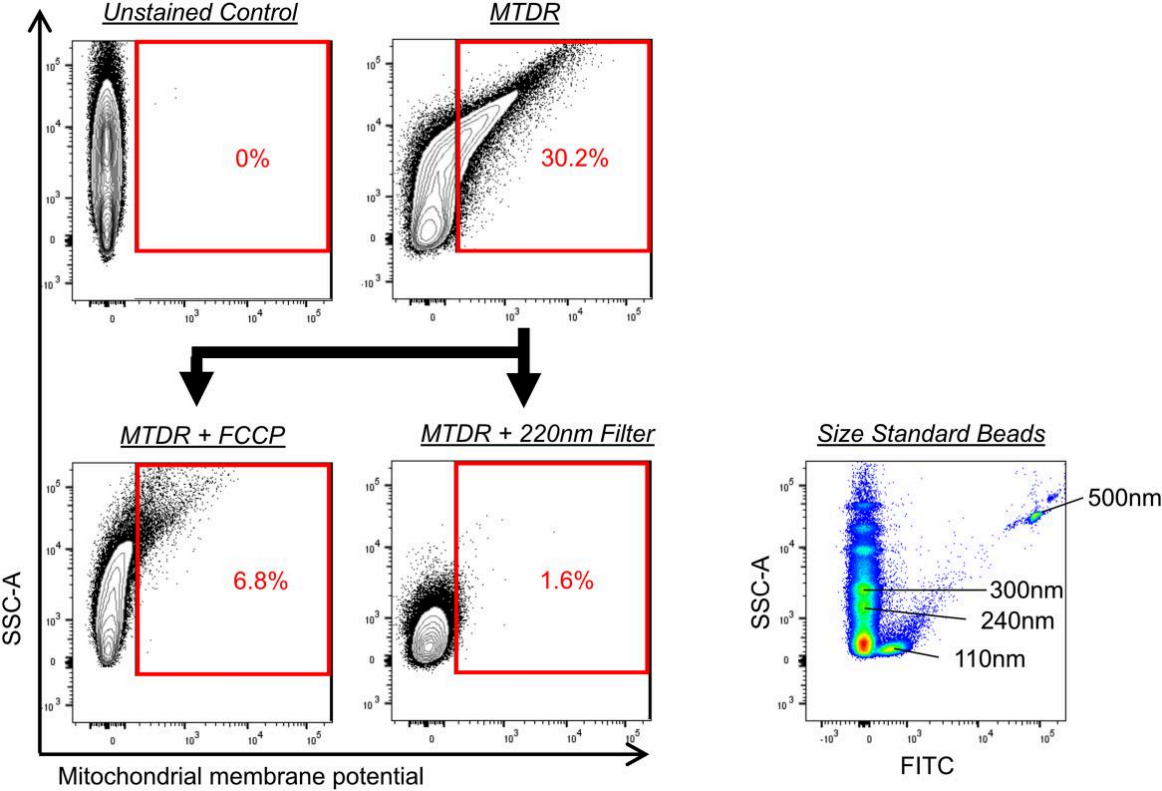
Representative flow cytometric dot plots of plasma extracellular vesicles (EVs) and size-standard beads. Plasma EVs were stained with MitoTracker Deep Red (MTDR), a mitochondrial membrane potential indicator. MitoTracker Deep Red fluorescence was diminished by carbonyl cyanide-p-trifluoromethoxyphenylhydrazone (FCCP), a mitochondrial uncoupler. In addition, filtration through 220 nm filters markedly reduced the proportion of EVs corresponding to diameters >240 nm, as calibrated by side scatter area (SSC-A) using size-standard beads.

Mitochondrial membrane potential of inflammatory and noninflammatory EV clusters was assessed using MTDR (Figure 3) [31–33]. Gating was based on the negative controls. To validate the specificity of MTDR detection by flow cytometry, 2 independent approaches were employed. First, plasma EVs were treated with carbonyl cyanide-p-trifluoromethoxyphenylhydrazone (FCCP 1.0 μM for 5 minutes), a protonophore that selectively dissipates mitochondrial membrane potential, which reduced the proportion of MTDR^+^ EVs from 30.2% to 6.8% (Figure 3). Second, consistent with previous reports indicating that mitochondria-containing EVs are >200 nm [31–33], we subjected plasma EVs to filtration through a 220 nm membrane. This procedure markedly reduced the proportion of MTDR^+^ EVs to 1.6% (Figure 3). Collectively, these results support the specificity of MTDR for detecting mitochondrial membrane potential-associated signals in plasma EVs.

### Statistical Analysis

Data are presented as boxplots with individual data points. For the pandemic-era cohort, statistical significance was determined using 1-way analysis of variance (ANOVA) with Tukey's multiple comparison test. For the prepandemic cohort, a 2-sided Student's *t*-test was used. Data distributions were visually inspected to assess suitability for parametric testing. To control for multiple testing, false discovery rate (FDR) correction was applied using the Benjamini–Hochberg procedure within each family of related outcomes (NTA metrics, surface EV markers, and mitochondrial membrane potential positivity). Pandemic-era and prepandemic cohorts were analyzed separately, with FDR correction applied independently within each cohort. Statistical significance after correction was defined as *q* < 0.05. To assess potential confounding by demographic and clinical variables, multivariable linear regression analyses were performed in the pandemic-era cohort for each EV marker and mitochondrial membrane potential subset, including study group, age, sex, and vaccination status as covariates. Due to incomplete covariate data, 1 participant from the LC-ME/CFS group and 1 participant from the pan-ME/CFS group were excluded from these adjusted analyses. Type II ANOVA was used to evaluate the global effect of each predictor within the fitted linear models. Covariate-adjusted analyses were not performed for the prepandemic cohort due to the unavailability of individual-level demographic and vaccination data. All analyses were performed using R (version 4.3.1) and GraphPad Prism (version 10.5.0).

## RESULTS

### Plasma EV Concentration is Increased in ME/CFS Groups

**Figure 4.**
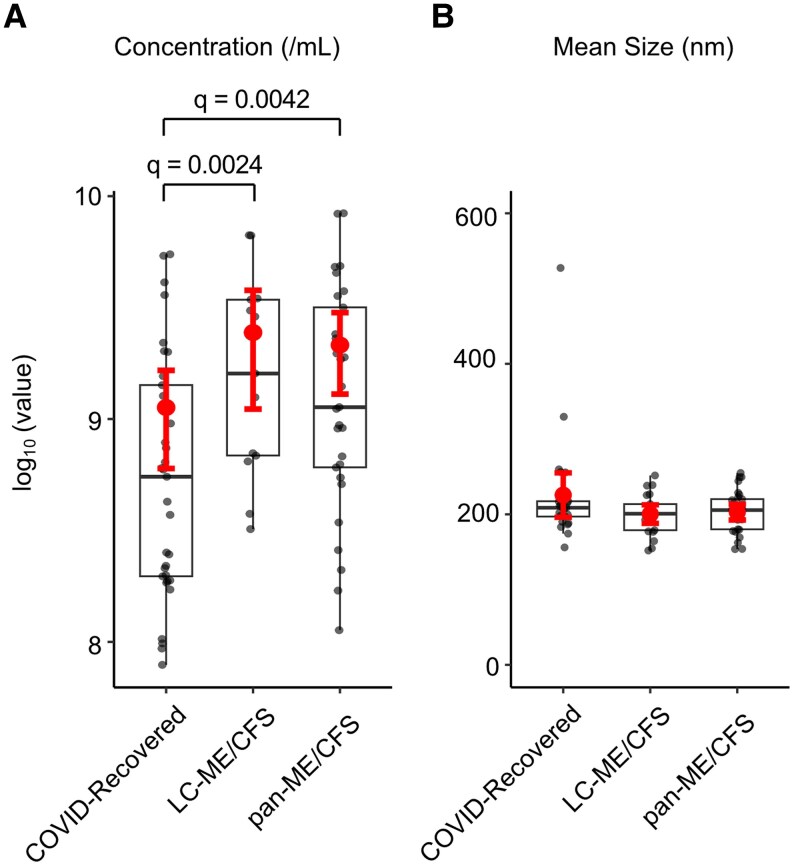
Nanoparticle tracking analysis of plasma extracellular vesicles (EVs) isolated from pandemic-era samples. *A*, Pandemic-era myalgic encephalomyelitis/chronic fatigue syndrome (ME/CFS) group (pan-ME/CFS) and long COVID with ME/CFS (LC-ME/CFS) exhibited a significantly higher EV concentration compared with the COVID-recovered group after false discovery rate (FDR) correction. *B*, No significant difference was observed in mean EV size among groups. Data are shown as boxplots with individual data points. Symbols indicate group means, and error bars denote 95% confidence interval. Statistical analyses were performed using 1-way analysis of variance with Tukey's post hoc test. FDR correction was applied using the Benjamini–Hochberg procedure, and adjusted *q*–value of <0.05 was considered statistically significant.

Nanoparticle tracking analysis was performed to evaluate plasma EV concentration and size across pandemic-era study cohorts. The mean EV concentrations were 4.12 × 10^8^ particles/mL in COVID-recovered individuals, 1.42 × 10^9^ particles/mL in the LC-ME/CFS group, and 1.23 × 10^9^ particles/mL in the pan-ME/CFS group (Figure 4*A*). Following log transformation, EV concentrations were significantly higher in both the pan-ME/CFS and LC-ME/CFS groups compared with COVID-recovered controls, whereas no difference was observed between the 2 ME/CFS groups. These differences remained significant after FDR correction (Figure 4*A* and Supplementary Table 2). In a multivariable linear regression model using log-transformed EV concentration and including age, sex, and vaccination status as covariates, diagnostic group remained significantly associated with EV concentration (type II ANOVA, *P* = .0047, Supplementary Table 3), indicating that the observed differences were not explained by these demographic variables. In contrast, no significant differences in mean EV size were observed among the 3 groups (COVID-recovered: 225 nm; LC-ME/CFS: 200 nm; pan-ME/CFS: 202 nm; Figure 4*B*).

### Flow Cytometric Profiling Reveals Distinct Cellular Origins of Plasma EVs in the LC-ME/CFS and Pan-ME/CFS

**Figure 5.**
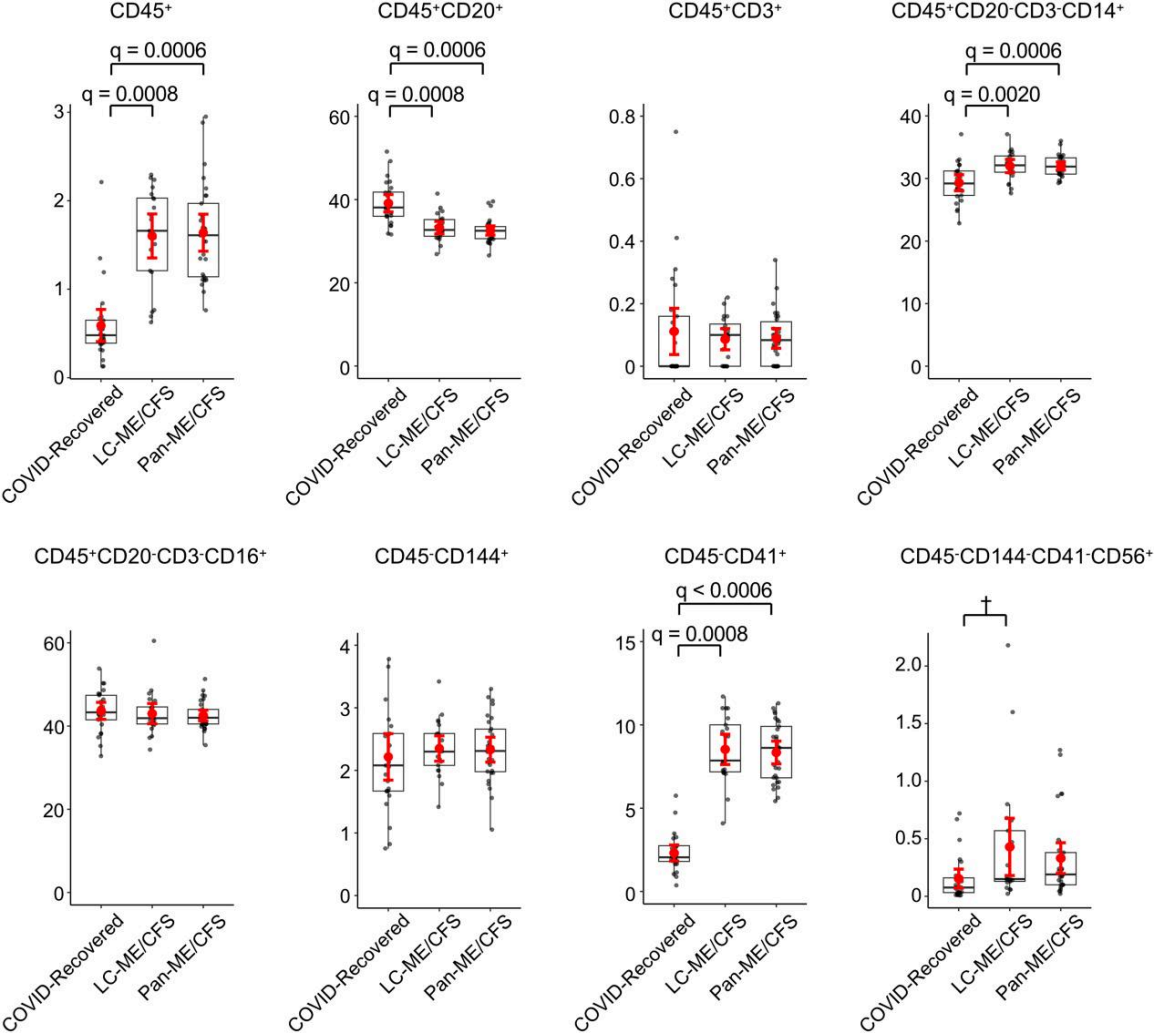
Flow cytometric analysis of plasma extracellular vesicles (EVs) surface marker expression. The percentages of EVs positive for immune and nonimmune cell surface markers were compared across 3 groups: COVID-recovered (n = 25), long COVID (LC) with myalgic encephalomyelitis/chronic fatigue syndrome (ME/CFS) phenotype (LC-ME/CFS, n = 21), and ME/CFS without prior COVID-19 (pan-ME/CFS, n = 29). Markers analyzed include: CD45 (leukocyte), CD20 (B cells), CD3 (T cells), CD14 (monocytes/macrophages), CD16 (NK cells), CD144 (endothelial cells), CD41 (platelets), and CD56 (neural cells). Data are shown as boxplots with individual data points. Symbols indicate group means, and error bars denote 95% confidence interval. Statistical analysis was performed using 1-way analysis of variance with Tukey's post hoc test. False discovery rate (FDR) correction was applied using the Benjamini–Hochberg procedure, and adjusted *q*-value of <0.05 was considered statistically significant. ^†^Nominal *P* < .05 but not significant after FDR.

To investigate the cellular origin of plasma EVs, surface marker profiling was performed using flow cytometry. Within the total EV population defined by forward and side scatter (FSC/SSC), the proportion of CD45^+^ EVs (leukocyte marker) was significantly increased in both LC-ME/CFS and pan-ME/CFS compared with the COVID-recovered group, and these differences remained significant after FDR correction (Figure 5 and Supplementary Table 4). Importantly, these associations remained statistically significant in multivariable linear models adjusting for age, sex, and vaccination status (Supplementary Table 3). Among CD45^+^ EVs, CD20^+^ EVs (B cell derived) were significantly reduced in the LC-ME/CFS and pan-ME/CFS compared with the COVID-recovered group, whereas CD3^+^ EVs (T cell derived) did not differ significantly across groups. Within the CD45^+^CD20^−^CD3^−^ EV subset (non-B, non-T leukocyte derived), CD14^+^ EVs (monocyte/macrophage derived) were significantly increased in both LC-ME/CFS and pan-ME/CFS groups compared with the COVID-recovered group, and this difference remained significant after FDR correction and covariate adjustment. In contrast, CD16^+^ EVs (NK cell derived) showed no significant group differences in either unadjusted or adjusted analyses. Among CD45^−^ EVs, CD144^+^ EVs (endothelial derived) showed no significant differences. However, CD41^+^ EVs (platelet derived) were significantly elevated in both LC-ME/CFS and pan-ME/CFS compared with the COVID-recovered group, and these differences remained significant after FDR correction and covariate adjustment. To explore potential nervous system involvement, we examined the CD45^−^CD144^−^CD41^−^CD56^+^ subset. Although this neural cell–associated population was detected at low levels overall, patients with LC-ME/CFS exhibited a nominal increase compared with the COVID-recovered group (*P* < .05); however, this difference did not remain statistically significant after FDR correction and was not significant in adjusted models. Collectively, these findings indicate selective enrichment of inflammatory immune- and platelet-associated EV subsets in the LC-ME/CFS and pan-ME/CFS, with no broad shift across all cellular compartments.

### Increased Mitochondrial Membrane Potential in Plasma EVs Derived From Inflammatory Cell Populations

**Figure 6.**
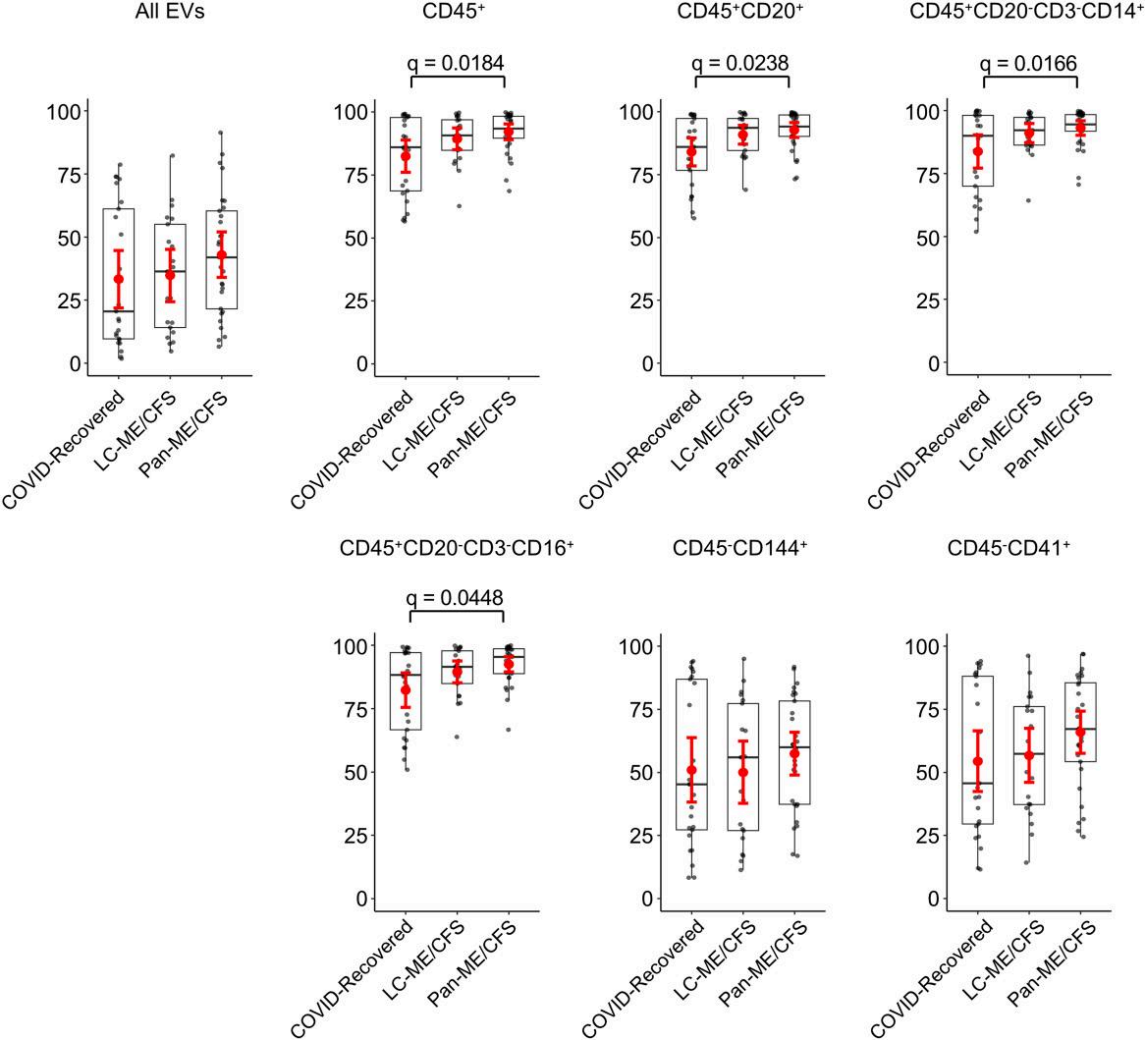
Mitochondrial membrane potential-positive extracellular vesicles (EVs) within cell type-specific plasma EV subsets. Plasma EVs were stained with a mitochondrial membrane potential marker (MitoTracker Deep Red), and the percentage of positive EVs was assessed in each surface marker-defined subset. Mitochondrial analysis was not conducted in CD45^+^CD3^+^ (T cell–derived) and CD45^−^CD144^−^CD41^−^CD56^+^ (neural-derived) EVs due to limited event numbers. Data are shown as boxplots with jittered individual values. Symbols indicate group means, and error bars denote 95% confidence interval. One-way analysis of variance with Tukey's post hoc test was used for statistical analysis. False discovery rate (FDR) correction was applied using the Benjamini–Hochberg procedure, and adjusted *q*-value of <0.05 was considered statistically significant.

Extracellular vesicles positive for mitochondrial membrane potential were quantified within each plasma EV cluster. In the total EV population defined by FSC/SSC, the mean proportion of EVs positive for mitochondrial membrane potential markers was 33.2% in the COVID-recovered group, 34.7% in the LC-ME/CFS group, and 43.0% in the pan-ME/CFS group, with no statistically significant differences among the groups (Figure 6). Across all CD45^+^ EV subsets examined (total leukocyte-, B cell–, monocyte/macrophage-, and NK cell–derived EV subsets), mitochondrial membrane potential positivity was significantly elevated in the pan-ME/CFS compared with the COVID-recovered group. These differences remained significant after FDR correction and in multivariable models adjusting for age, sex, and vaccination status (Supplementary Tables 2 and 5). No significant differences were observed in CD144^+^ (endothelial-derived) or CD41^+^ (platelet-derived) EVs. Due to limited event counts, mitochondrial membrane potential analyses could not be performed for CD45^+^CD3^+^ (T cell–derived) and CD45^−^CD144^−^CD41^−^CD56^+^ (neural-derived) EV subsets. Collectively, these findings indicate that increased proportions of mitochondrial membrane potential-positive EVs are selectively enriched within inflammatory immune-derived EV subsets rather than reflecting a global alteration across all circulating EV populations.

### Plasma EV Concentration and Size Distribution in the Prepandemic Cohort

**Figure 7.**
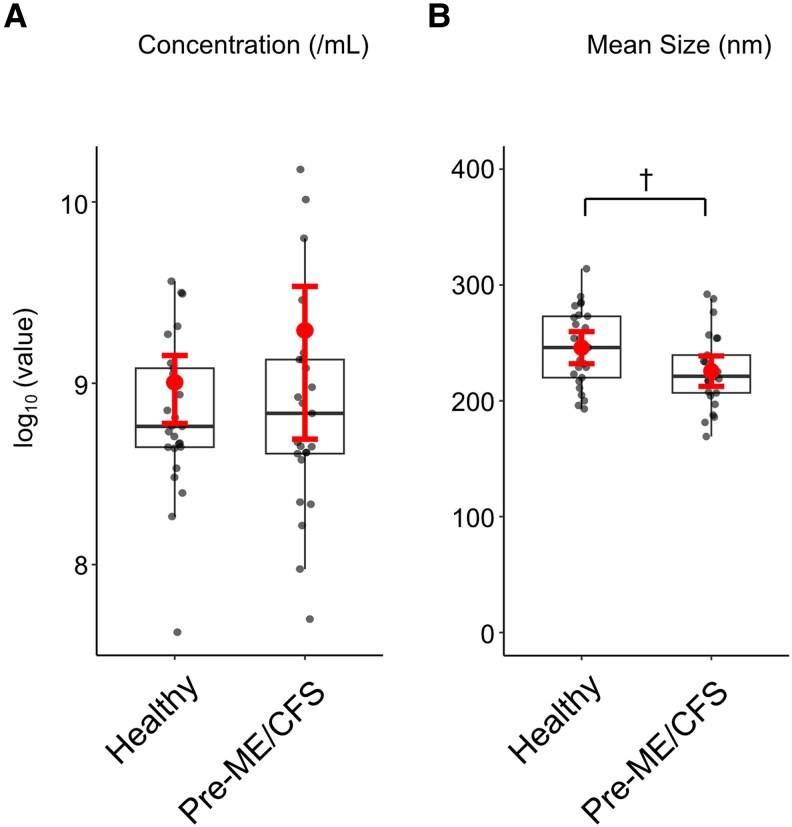
Concentration and mean size of plasma extracellular vesicles (EVs) isolated from prepandemic samples. *A*, No significant difference in EV concentration was detected between groups. *B*, The mean EV size was significantly smaller in the prepandemic myalgic encephalomyelitis/chronic fatigue syndrome (pre-ME/CFS) group compared with the healthy group. Data are shown as boxplots with individual data points. Symbols indicate group means, and error bars denote 95% confidence interval. Statistical analyses were performed using 2-sided Student's *t*-test for prepandemic samples. False discovery rate (FDR) correction was applied using the Benjamini–Hochberg procedure, and adjusted *q*-value of <0.05 was considered statistically significant. ^†^Nominal *P* < .05 but not significant after FDR.

To evaluate plasma EV characteristics independent of SARS-CoV-2 infection or vaccination, we analyzed samples from a prepandemic cohort comprising patients with ME/CFS (pre-ME/CFS) and matched healthy controls. Extracellular vesicle concentrations did not differ significantly between groups after log transformation (Figure 7*A*). Mean EV diameter was lower in the pre-ME/CFS group compared with healthy controls (225 nm vs 248 nm; nominal *P* = .0331, Figure 7*B*). However, this difference did not remain statistically significant after FDR correction (Supplementary Table 6). Collectively, these findings indicate no significant alterations in global EV concentration or size distribution after multiple testing correction.

### Surface Marker Profiling of Plasma EVs in the Prepandemic Cohort

**Figure 8.**
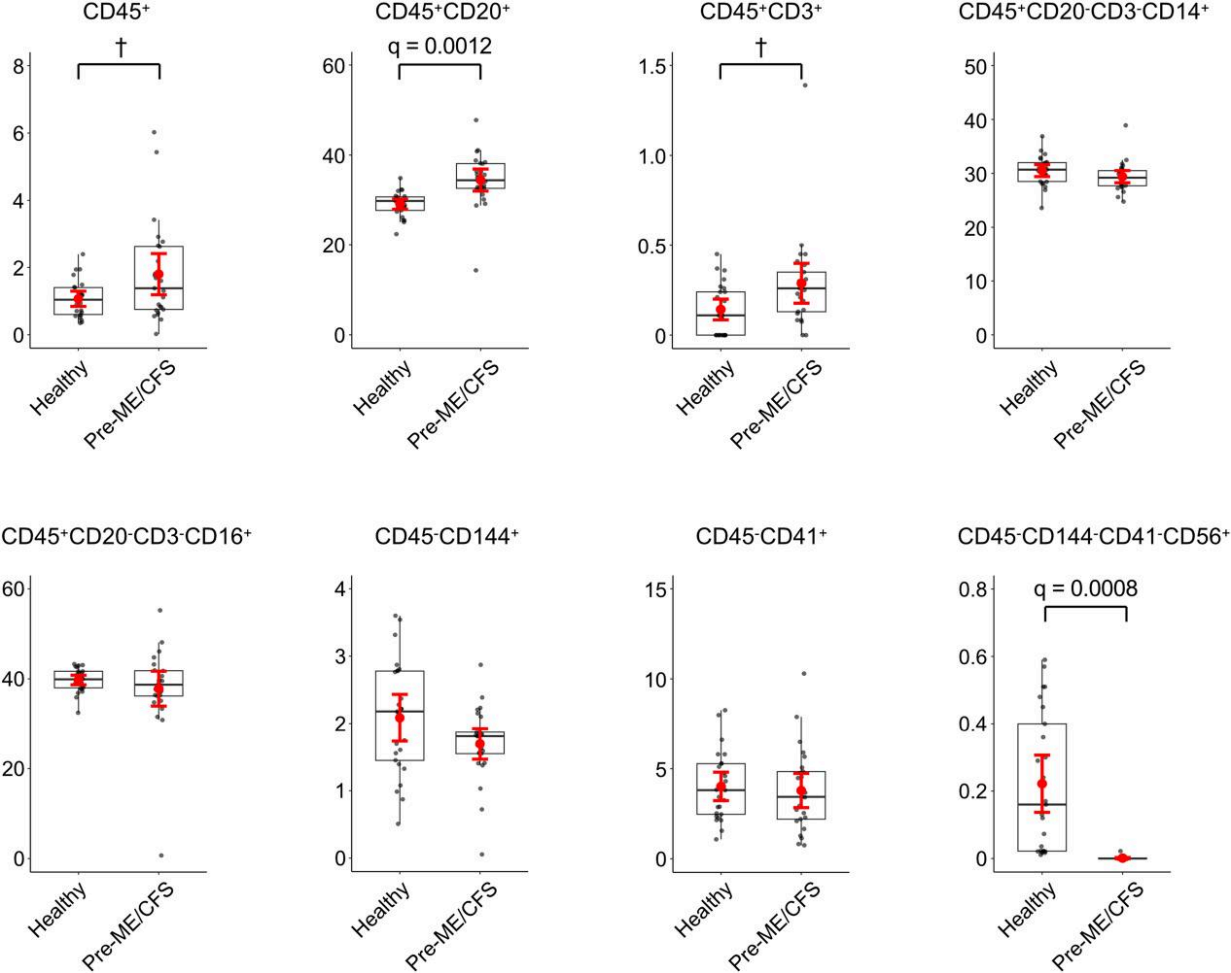
Comparison of surface marker expression on plasma extracellular vesicles (EVs) between healthy and prepandemic myalgic encephalomyelitis/chronic fatigue syndrome (pre-ME/CFS) groups. The percentages of EVs positive for immune and nonimmune cell surface markers were compared between pre-ME/CFS (n = 25) and healthy controls (n = 25). Markers analyzed include: CD45 (leukocyte), CD20 (B cells), CD3 (T cells), CD14 (monocytes/macrophages), CD16 (NK cells), CD144 (endothelial cells), CD41 (platelets), and CD56 (neural cells). Data are shown as boxplots with individual data points. Symbols indicate group means, and error bars denote 95% confidence interval. Statistical analysis was performed using a 2-sided Student's *t*-test. False discovery rate (FDR) correction was applied using the Benjamini–Hochberg procedure, and adjusted *q*-value of <0.05 was considered statistically significant. ^†^Nominal *P* < .05 but not significant after FDR.

Surface marker profiling was also performed in the prepandemic cohort. Within the total EV population, the proportion of CD45^+^ EVs was significantly higher in patients with pre-ME/CFS compared with healthy controls; however, this difference did not persist after FDR adjustment (Figure 8 and Supplementary Table 7). Among CD45^+^ EVs, CD20^+^ (B cell–derived) EVs were significantly increased in pre-ME/CFS, and this association remained significant after FDR correction. Although CD3^+^ EVs (T cell derived) were present at low levels in both groups, their proportion was significantly higher in the pre-ME/CFS; however, this difference did not persist after FDR adjustment. Within the CD45^+^CD20^−^CD3^−^ subset, no significant differences were observed in CD14^+^ (monocyte/macrophage-derived) or CD16^+^ (NK cell–derived) EVs between groups. Among CD45^−^ EV subsets, the proportions of CD144^+^ (endothelial-derived) and CD41^+^ (platelet-derived) EVs did not differ between pre-ME/CFS and healthy controls. Within the CD45^−^CD144^−^CD41^−^ subset, CD56^+^ EVs (neural derived) were present at low levels in both groups but were significantly reduced in patients with pre-ME/CFS, and this difference remained significant after FDR correction. Collectively, these findings indicate that EV alterations in the prepandemic cohort were limited to specific leukocyte- and neural-associated subsets rather than reflecting widespread changes across immune or vascular EV compartments.

### Mitochondrial Membrane Potential of Plasma EVs in the Prepandemic Cohort

**Figure 9.**
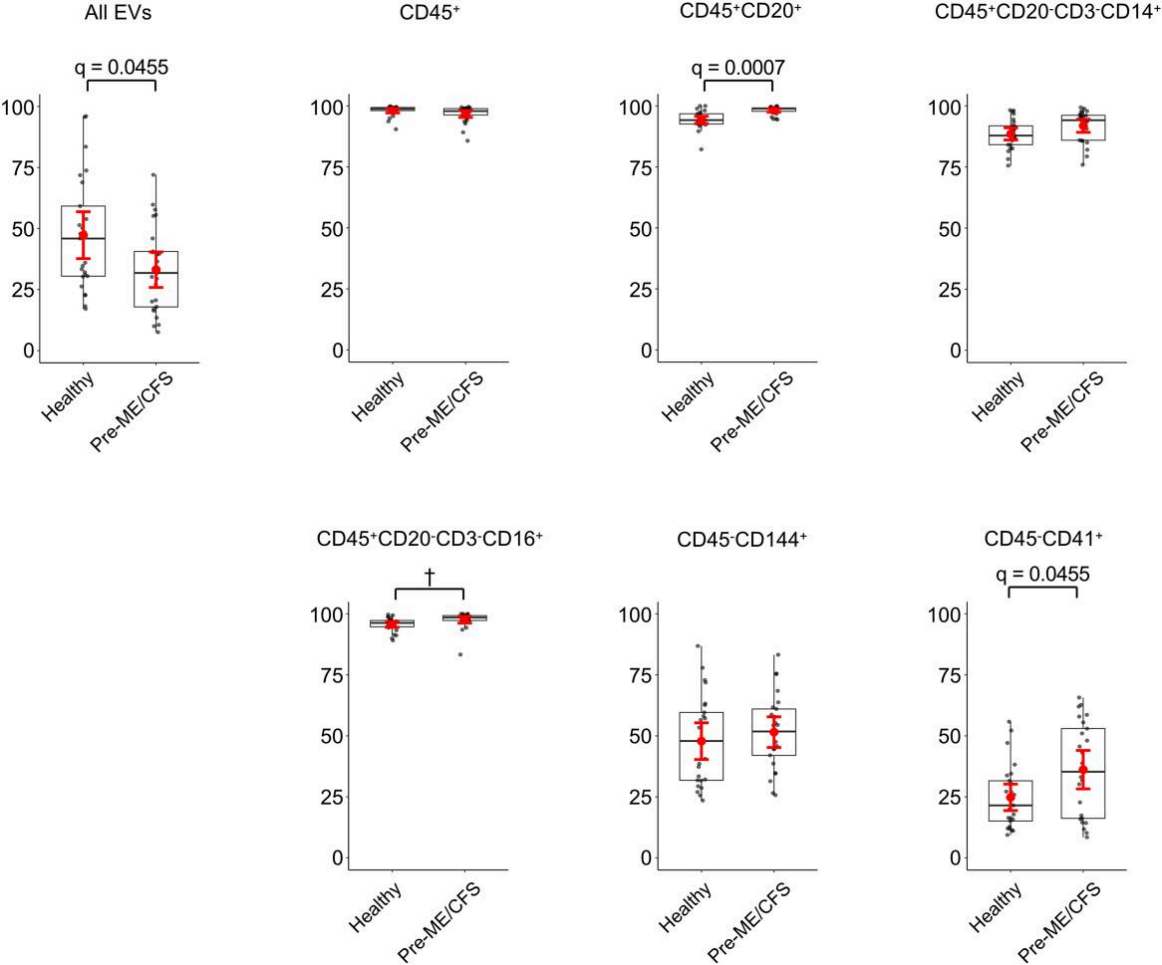
Mitochondrial marker-positive extracellular vesicles (EVs) in plasma from Healthy and prepandemic myalgic encephalomyelitis/chronic fatigue syndrome (pre-ME/CFS) subjects. Extracellular vesicles were stained with a mitochondrial membrane potential dye (MitoTracker Deep Red), and the percentage of positive EVs was measured in total EVs and cell type-specific subsets defined by surface markers. Mitochondrial analysis was not conducted in CD45^+^CD3^+^ (T cell–derived) and CD45^−^CD144^−^CD41^−^CD56^+^ (neural-derived) EVs due to limited event numbers. Data are shown as boxplots with individual data points. Symbols indicate group means, and error bars denote 95% confidence interval. Statistical analysis was performed using a 2-sided Student's *t*-test. False discovery rate (FDR) correction was applied using the Benjamini–Hochberg procedure, and adjusted *q*-value of <0.05 was considered statistically significant. ^†^Nominal *P* < .05 but not significant after FDR.

We assessed the proportion of mitochondrial membrane potential-positive EVs within defined subpopulations in the prepandemic cohort. At the total EV level, healthy controls exhibited significantly higher mitochondrial membrane potential positivity compared with the pre-ME/CFS, and this difference remained significant after FDR correction (Figure 9 and Supplementary Table 8). Although overall mitochondrial positivity within total CD45^+^ inflammatory EVs did not differ between groups, CD45^+^CD20^+^ (B cell–derived) EVs exhibited significantly higher mitochondrial membrane potential positivity in the pre-ME/CFS compared with healthy controls, and this association remained significant after FDR correction. In the CD45^+^CD20^−^CD3^−^ population, mitochondrial membrane potential positivity in CD14^+^ (monocyte/macrophage-derived) EVs did not differ between groups. CD16^+^ (NK cell–derived) EVs showed a nominal increase in the pre-ME/CFS; however, this difference did not persist after FDR adjustment. Among CD45^−^ EV subsets, mitochondrial membrane potential positivity in CD144^+^ (endothelial-derived) EVs was comparable between groups. In contrast, CD45^−^CD41^+^ (platelet-derived) EVs exhibited significantly higher mitochondrial membrane potential positivity in pre-ME/CFS compared with healthy controls, and the difference remained significant after FDR correction. Mitochondrial membrane potential analyses were not performed in CD45^+^CD3^+^ (T cell–derived) and CD45^−^CD144^−^CD41^−^CD56^+^ (neural-derived) EV subsets due to limited event numbers. Overall, these findings indicate a heterogeneous distribution of mitochondrial membrane potential across EV subpopulations in the prepandemic cohort, without a uniform directional shift across all subsets.

## DISCUSSION

Although there is consensus regarding the clinical diagnosis of LC (NASEM, CDC, NICE, WHO, and others), and multiple hypotheses, including micro-clots, endothelial dysfunction, dysbiosis, immune dysregulation, mitochondrial dysfunction, and viral persistence, have been proposed, the identification of robust biological markers remains elusive. Extracellular vesicles, which mediate intercellular communication under physiological and pathological conditions, represent promising biomarker candidates [45]. Given the marked clinical heterogeneity of LC, with over 200 reported symptoms across multiple clusters and phenotypes, including ME/CFS and postural tachycardia syndrome [46, 47], we focused on a clinically homogeneous subgroup fulfilling ME/CFS diagnostic criteria, along with an LC cohort with prolonged symptom duration.

Nanoparticle tracking analysis and flow cytometric profiling revealed shared EV alterations between LC-ME/CFS and pan-ME/CFS. In the pandemic-era cohort, LC-ME/CFS and pan-ME/CFS demonstrated increased plasma EV concentrations with enrichment of inflammatory immune-derived EVs, particularly CD45^+^ leukocyte, CD45^+^CD14^+^ monocyte/macrophage, and platelet subsets. These findings are consistent with previously described immunophenotypes [21, 47] and align with several pathobiological hypotheses of LC, including platelets/micro-clots [48], immune cell disturbances involving B cells [49], and monocyte activation [50]. No significant changes were observed in endothelial-derived (CD144^+^) EV subsets. Elevated mitochondrial membrane potential positivity was observed within EV subsets derived from B cells, monocytes/macrophages, and NK cells in pan-ME/CFS, suggesting enhanced activation of inflammatory immune cell–derived EVs [21]. Importantly, no differences were observed at the level of total EV mitochondrial membrane potential in the pan-ME/CFS cohort, indicating that the alteration reflects a selective immune cell–associated phenomenon rather than a global shift in EV mitochondrial content. In contrast, no statistically significant differences in mitochondrial membrane potential were observed across immune cell-, endothelial-, or platelet-derived EV subsets in the LC-ME/CFS cohort, suggesting these mitochondrial membrane potential alterations are preferentially observed in pan-ME/CFS.

When comparing ME/CFS across cohorts, these compositional patterns were not consistently observed. The only mitochondrial signal that consistently remained significant after FDR correction was observed in the CD45^+^CD20^+^ (B cell–derived) EV subset, whereas other immune-derived subsets demonstrated nominal or cohort-dependent differences that did not uniformly persist after multiple testing correction. These findings suggest that while immune-associated EV alterations may recur across cohorts, the specific pattern of EV subset enrichment varies according to biological context and cohort conditions. CD56^+^ EVs represented a relatively low-abundance subset and demonstrated divergent directional patterns across cohorts; therefore, these observations should be interpreted cautiously. Importantly, EV subsets were quantified as hierarchical proportions rather than normalized absolute counts, and observed differences may reflect compositional shifts within leukocyte-derived compartments rather than definitive alterations in absolute EV release. Surface marker–based phenotyping may be influenced by membrane shedding, trogocytosis, or receptor exchange [51, 52]. Accordingly, our findings should be interpreted as relative enrichment of lineage-associated EV subsets rather than definitive proof of cellular origin.

Comparison with prior studies revealed partial concordance with our findings. In our previous work [53], we reported alterations in platelet-associated and B cell–associated EV subsets in ME/CFS. Consistent with this, we observed enrichment of CD45^+^ EVs in the pandemic-era cohort. However, in contrast to earlier findings, total EV concentration was increased in the pandemic-era cohort, suggesting context-dependent modulation of circulating EV abundance. Recent proteomic studies of exercise-responsive EVs in ME/CFS demonstrated postexertional metabolic alterations within EV cargo [54]. In contrast, our findings identify baseline alterations in mitochondrial membrane potential within defined immune subsets, indicating that immune cell–associated mitochondrial EV features may be present independent of acute exertion. Prior LC studies have reported increased immune marker–positive and platelet-derived EV populations, consistent with our pandemic-era cohort [55]. However, these studies primarily emphasized EV cargo-mediated mitochondrial dysfunction in recipient cells. In contrast, our data demonstrate alterations in mitochondrial membrane potential within circulating immune-derived EV subsets, providing complementary evidence that mitochondrial-associated EV abnormalities may originate within immune cell–derived vesicles.

The divergent compositional patterns between cohorts may reflect cohort-specific biological context rather than technical artifacts. First, prepandemic samples underwent longer cryostorage, which may affect EV integrity or epitope stability [56]. Second, differences in disease duration or severity could have contributed, as patients in the pre-ME/CFS cohort may have had a longer history of illness. Third, patients with pan-ME/CFS may have been exposed to additional immunological stimuli, including overt or subclinical SARS-CoV-2 infection, vaccination, or pandemic-associated lifestyle changes, which could have modulated immune cell function and mitochondrial metabolism.

The enrichment of CD45^+^ EVs is consistent with previous reports demonstrating that inflammatory stimuli enhance EV release from immune cells [57–59]. In both cohorts, increased mitochondrial membrane potential positivity was observed within B cell–derived EV subsets. Mitochondrial membrane potential reflects mitochondrial polarization and metabolic state; thus, enrichment within immune-derived EVs may indicate altered immune cell bioenergetic characteristics in ME/CFS-related conditions [60–64]. Alternative mechanisms include altered mitophagy or selective incorporation of mitochondrial components under inflammatory stress [65–67]. In addition, mitochondrial hyperpolarization can occur as a compensatory response in metabolically stressed or dysfunctional cells [68, 69]. Because parent-cell metabolic flux and activation states were not directly assessed, mechanistic interpretation remains inferential.

In conclusion, inflammatory immune cell–derived plasma EVs exhibit context-dependent alterations in ME/CFS and LC-ME/CFS. Within the pandemic-era cohort, LC-ME/CFS and pan-ME/CFS demonstrated broadly comparable EV compositional features, whereas mitochondrial membrane potential alterations were more prominent in pan-ME/CFS. Across cohorts, mitochondrial membrane potential alterations were most robustly observed in B cell–derived EV subsets, although the overall pattern varied by cohort. These findings suggest that alterations in mitochondrial membrane potential within specific immune cell–derived EV populations may occur in ME/CFS-related conditions and could inform future biomarker development.

### Limitations

This study has several limitations. First, its cross-sectional design precludes causal inference. Second, recruitment structures differed between cohorts, and sex distribution imbalance may affect generalizability despite multivariable adjustment. Vaccination status was included as a covariate; however, subgroup analyses by infection or vaccination status were not performed due to limited sample size. Third, the study was not powered a priori for subset-specific comparisons, and nonsignificant findings may reflect limited statistical power. Standardized severity and functional impairment measures were not uniformly available, limiting evaluation of EV signatures in relation to disease burden. In the prepandemic cohort, lack of individual-level covariate data precluded adjusted analyses. Fourth, technical considerations also apply. Flow cytometry does not permit definitive assignment of EV cellular origin, and dye-based mitochondrial membrane potential assessment remains an emerging approach subject to signal sensitivity and dye-loading variability. Extracellular vesicle subsets were quantified as proportions rather than absolute counts. Long-term cryostorage may additionally influence EV integrity. Finally, because cohorts were collected at different time points without prospective harmonization, broader temporal or postviral influences cannot be excluded. Accordingly, future longitudinal, prospectively harmonized, and rigorously designed hypothesis-driven studies integrating single-EV technologies, high-resolution imaging, bioenergetic assays, and multi-omics approaches will be essential to confirm causality and clarify mechanistic relationships.

## Supplementary Material

ofag209_Supplementary_Data

## Supplementary Data

Supplementary materials are available at *Open Forum Infectious Diseases* online. Consisting of data provided by the authors to benefit the reader, the posted materials are not copyedited and are the sole responsibility of the authors, so questions or comments should be addressed to the corresponding author.
