# Supplementary material for: Plasma Extracellular Vesicle Surface Marker Profiling Reveals Immune Cell–Associated Mitochondrial Membrane Potential Alterations in Long COVID and Myalgic Encephalomyelitis/Chronic Fatigue Syndrome
Source: Open Forum Infect Dis. 2026 May 12;13(5):ofag209. doi: 10.1093/ofid/ofag209 (PMC13166156; doi:10.1093/ofid/ofag209)
Supplement: ofag209_Supplementary_Data [file ofag209_supplementary_data.docx]

**Supplementary Table 1. Antibody Information**

| **Antibody** | **Dilution** | **Manufacturer** | **Catalog No.** |
| --- | --- | --- | --- |
| Mouse Anti-Human CD45 | 1:1,000 | BD Biosciences | 564105 |
| Mouse Anti-Human CD20  Mouse Anti-Human CD3  Mouse Anti-Human CD14  Mouse Anti-Human CD16  Mouse Anti-Human CD144  Mouse Anti-Human CD41a  Mouse Anti-Human CD56  Human IgG1, κ Isotype Control  Mouse IgG1, κ Isotype Control  Mouse IgG1, κ Isotype Control  Mouse IgG1, κ Isotype Control  Mouse IgG1, κ Isotype Control  Mouse IgG1, κ Isotype Control  Mouse IgG2a, κ Isotype Control  Mouse IgG2b, κ Isotype Control | 1:1,000  1:1,000  1:1,000  1:1,000  1:1,000  1:1,000  1:1,000  1:1,000  1:1,000  1:1,000  1:1,000  1:1,000  1:1,000  1:1,000  1:1,000 | BD Biosciences  BD Biosciences  Thermo Fisher Scientific  BD Biosciences  Miltenyi Biotec  BD Biosciences  Thermo Fisher Scientific  Miltenyi Biotec  Thermo Fisher Scientific  BD Biosciences  BD Biosciences  BD Biosciences  BD Biosciences  BD Biosciences  BD Biosciences | 612905  564713  78-0149-42  740833  130-100-720  559768  11-0566-42  130-128-392  78-4714-82  550795  563044  340755  555750  563927  612907 |

**Supplementary Table 2. Statistical comparisons and FDR-adjusted results for NTA metrics in the pandemic-era cohort**

|  | **COVID-Recovered vs. LC-ME/CFS** | | | **COVID-Recovered vs. ME/CFS** | | | **LC-ME/CFS vs. ME/CFS** | | |
| --- | --- | --- | --- | --- | --- | --- | --- | --- | --- |
|  | **P value** | **FDR** | **Significance** | **P value** | **FDR** | **Significance** | **P value** | **FDR** | **Significance** |
| Concentration | 0.0012 | 0.0024 | Yes | 0.0021 | 0.0042 | Yes | 0.8946 | 0.9787 | No |
| Mean size | 0.1723 | 0.1723 | No | 0.1905 | 0.1905 | No | 0.9787 | 0.9787 | No |

P values were derived from one-way ANOVA with Tukey post hoc testing. False discovery rate (FDR) correction was applied using the Benjamini–Hochberg procedure.

|  | **Study group** | | **Sex** | | **Age** | | **Vaccination Status** | |
| --- | --- | --- | --- | --- | --- | --- | --- | --- |
|  | **F value** | **P value** | **F value** | **P value** | **F value** | **P value** | **F value** | **p value** |
| NTA concentration (log scale) | 5.79 | 0.0047 | 1.43 | 0.2346 | 0.81 | 0.3709 | 0.04 | 0.8241 |
| CD45^+^ EVs | 24.38 | <0.0001 | 0.53 | 0.4685 | 0.12 | 0.7256 | 2.00 | 0.1617 |
| CD20^+^ EVs | 6.32 | 0.0030 | 1.44 | 0.2346 | 0.50 | 0.4818 | 0.43 | 0.5136 |
| CD14^+^ EVs | 4.48 | 0.0148 | 0.04 | 0.8453 | 0.00 | 0.9820 | 0.00 | 0.9463 |
| CD41^+^ EVs | 65.60 | <0.0001 | 0.29 | 0.5949 | 0.01 | 0.9129 | 3.17 | 0.0794 |
| Mito positivity in CD45^+^ EVs | 5.06 | 0.0089 | 0.96 | 0.3303 | 0.33 | 0.5670 | 1.29 | 0.2606 |
| Mito positivity in CD20^+^ EVs | 4.54 | 0.0141 | 0.67 | 0.4170 | 0.30 | 0.5881 | 0.79 | 0.3775 |
| Mito positivity in CD14^+^ EVs | 4.85 | 0.0108 | 0.84 | 0.3625 | 0.04 | 0.8486 | 1.16 | 0.2852 |
| Mito positivity in CD16^+^ EVs | 5.34 | 0.0070 | 1.23 | 0.2703 | 0.18 | 0.6667 | 1.20 | 0.2766 |

**Supplementary Table 3. Multivariable ANOVA assessing the effects of study group, sex, age, and vaccination status on EV surface markers and mitochondrial positivity in the pandemic-era cohort**

|  | **COVID-Recovered vs. LC-ME/CFS** | | | **COVID-Recovered vs. ME/CFS** | | | **LC-ME/CFS vs. ME/CFS** | | |
| --- | --- | --- | --- | --- | --- | --- | --- | --- | --- |
|  | **P value** | **FDR** | **Significance** | **P value** | **FDR** | **Significance** | **P value** | **FDR** | **Significance** |
| CD45 | <0.0001 | 0.0008 | Yes | <0.0001 | 0.0006 | Yes | 0.9664 | 0.9999 | No |
| CD20 | <0.0001 | 0.0008 | Yes | <0.0001 | 0.0006 | Yes | 0.8104 | 0.9999 | No |
| CD3 | 0.7499 | 0.8739 | No | 0.7971 | 0.7971 | No | 0.9898 | 0.9999 | No |
| CD14 | 0.0010 | 0.0020 | Yes | 0.0003 | 0.0006 | Yes | 0.9999 | 0.9999 | No |
| CD16 | 0.8739 | 0.8739 | No | 0.638 | 0.7971 | No | 0.9341 | 0.9999 | No |
| CD144 | 0.7708 | 0.8739 | No | 0.7967 | 0.7971 | No | 0.9946 | 0.9999 | No |
| CD41 | <0.0001 | 0.0008 | Yes | <0.0001 | 0.0006 | Yes | 0.9198 | 0.9999 | No |
| CD56 | 0.0431 | 0.0689 | No† | 0.2052 | 0.32832 | No | 0.6429 | 0.9999 | No |

**Supplementary Table 4. Statistical comparisons and FDR-adjusted results for surface EV markers in the pandemic-era cohort**

P values were derived from one-way ANOVA with Tukey post hoc testing. False discovery rate (FDR) correction was applied using the Benjamini–Hochberg procedure. †Nominally significant before FDR but not significant after FDR correction.

**Supplementary Table 5. Statistical comparisons and FDR-adjusted results for mitochondrial membrane potential positivity in the pandemic-era cohort**

|  | **COVID-Recovered vs. LC-ME/CFS** | | | **COVID-Recovered vs. ME/CFS** | | | **LC-ME/CFS vs. ME/CFS** | | |
| --- | --- | --- | --- | --- | --- | --- | --- | --- | --- |
|  | **P value** | **FDR** | **Significance** | **P value** | **FDR** | **Significance** | **P value** | **FDR** | **Significance** |
| All EVs | 0.9789 | 0.9922 | No | 0.3323 | 0.3876 | No | 0.4828 | 0.8096 | No |
| CD45-positive | 0.1126 | 0.1970 | No | 0.0079 | 0.0184 | Yes | 0.6743 | 0.8096 | No |
| CD20-positive | 0.0660 | 0.1970 | No | 0.0068 | 0.0238 | Yes | 0.7962 | 0.8096 | No |
| CD14-positive | 0.0796 | 0.1970 | No | 0.0095 | 0.0166 | Yes | 0.8096 | 0.8096 | No |
| CD16-positive | 0.1058 | 0.1970 | No | 0.0064 | 0.0448 | Yes | 0.6514 | 0.8096 | No |
| CD144-positive | 0.9922 | 0.9922 | No | 0.6515 | 0.6515 | No | 0.6006 | 0.8096 | No |
| CD41-positive | 0.9479 | 0.9922 | No | 0.2183 | 0.3056 | No | 0.4099 | 0.8096 | No |

P values were derived from one-way ANOVA with Tukey post hoc testing. False discovery rate (FDR) correction was applied using the Benjamini–Hochberg procedure. **Supplementary Table 6. Statistical comparisons and FDR-adjusted results for NTA metrics in the pre-pandemic cohort**

|  | **Healthy vs. ME/CFS** | | |
| --- | --- | --- | --- |
|  | **P value** | **FDR** | **Significance** |
| Concentration | 0.7181 | 0.7181 | No |
| Mean size | 0.0331 | 0.0662 | No† |

P values were derived from one-way ANOVA with Tukey post hoc testing. False discovery rate (FDR) correction was applied using the Benjamini–Hochberg procedure. †Nominally significant before FDR but not significant after FDR correction.

**Supplementary Table 7. Statistical comparisons and FDR-adjusted results for surface EV markers in the pre-pandemic cohort**

|  | **Healthy vs. ME/CFS** | | |
| --- | --- | --- | --- |
|  | **P value** | **FDR** | **Significance** |
| CD45 | 0.0276 | 0.0552 | No† |
| CD20 | 0.0003 | 0.0012 | Yes |
| CD3 | 0.0214 | 0.0552 | No† |
| CD14 | 0.1510 | 0.2013 | No |
| CD16 | 0.3326 | 0.3801 | No |
| CD144 | 0.0607 | 0.0971 | No |
| CD41 | 0.7012 | 0.7012 | No |
| CD56 | <0.0001 | 0.0008 | Yes |

P values were derived from one-way ANOVA with Tukey post hoc testing. False discovery rate (FDR) correction was applied using the Benjamini–Hochberg procedure. †Nominally significant before FDR but not significant after FDR correction.

**Supplementary Table 8. Statistical comparisons and FDR-adjusted results for mitochondrial membrane potential positivity in the pre-pandemic cohort**

|  | **Healthy vs. ME/CFS** | | |
| --- | --- | --- | --- |
|  | **P value** | **FDR** | **Significance** |
| All EVs | 0.0195 | 0.0455 | Yes |
| CD45-positive | 0.1068 | 0.1246 | No |
| CD20-positive | <0.0001 | 0.0007 | Yes |
| CD14-positive | 0.0683 | 0.0956 | No |
| CD16-positive | 0.0330 | 0.0577 | No† |
| CD144-positive | 0.4414 | 0.4414 | No |
| CD41-positive | 0.0187 | 0.0455 | Yes |

P values were derived from one-way ANOVA with Tukey post hoc testing. False discovery rate (FDR) correction was applied using the Benjamini–Hochberg procedure. †Nominally significant before FDR but not significant after FDR correction.
